# Transcriptome Sequencing-Based Analysis of Premature Fruiting in *Amomum villosum* Lour.

**DOI:** 10.3390/biology14070883

**Published:** 2025-07-18

**Authors:** Yating Zhu, Shuang Li, Hongyou Zhao, Qianxia Li, Yanfang Wang, Chunyong Yang, Ge Li, Yanqian Wang, Lixia Zhang

**Affiliations:** 1 Yunnan Key Laboratory of Southern Medicinal Utilization, Yunnan Branch of Institute of Medicinal Plant Development, Chinese Academy of Medical Sciences and Peking Union Medical College, Jinghong 666100, China; 2 Institute of Medicinal Plant Development, Chinese Academy of Medical Sciences and Peking Union Medical College, Beijing 100193, China; 3 Yunnan Key Laboratory of Sustainable Utilization of Southern Medicine, Yunnan University of Traditional Chinese Medicine, Kunming 650500, China

**Keywords:** *Amomum villosum* Lour., premature fruiting, RNA-seq, differentially expressed gene

## Abstract

*Amomum villosum* Lour., a perennial medicinal plant in the Zingiber genus, usually requires approximately 3–4 years of vegetative growth from seed germination to first fruiting, resulting in high initial investment costs and a prolonged revenue cycle, which pose significant challenges to the industry’s sustainable development. Our research team observed a distinct premature fruiting phenomenon in *A. villosum*. We investigated the regulatory mechanisms underlying premature fruiting in *A. villosum* by identifying the key differentially expressed genes (DEGs) and metabolic pathways governing the premature fruiting (Precocious) and typical plants (CK) of the ‘Yunsha No.8’ cultivar. Transcriptomic sequencing (RNA-seq) and bioinformatic analyses were performed using the DNBSEQ^TM^ platform. The sequencing generated 29.0 gigabases (Gb) of clean data, and 115,965 unigenes were identified, with an average length of 1368 bp. Based on the sequencing results, 1545 DEGs were identified. Gene ontology (GO) analysis and Kyoto Encyclopedia of Genes and Genomes (KEGG) pathway analysis were annotated for these DEGs. This study identifies phytohormone signaling, carbohydrate and lipid metabolism, and polysaccharide degradation as critical pathways controlling premature fruiting in *A. villosum*. Six randomly selected DEGs were validated using real-time fluorescence quantitative polymerase chain reaction (RT-qPCR), and the results corroborated the transcriptome data, confirming their reliability. This study lays the foundation for the elucidation of the molecular mechanisms and metabolic pathways driving premature fruiting in *A. villosum*.

## Simple Summary

Our research team identified a distinct premature fruiting phenomenon in *Amomum villosum* Lour., which presents a promising opportunity to address the delayed economic returns commonly experienced during the cultivation of this crop. We investigated the regulatory mechanisms underlying premature fruiting in *Amomum villosum* Lour. with transcriptomic sequencing. The results suggest that the phytohormone signaling, carbohydrate and lipid metabolism, and polysaccharide degradation pathways are critical in controlling premature fruiting in *A. villosum*. These results enrich the understanding of the basic biology of this species, lay a solid foundation for the study of the molecular mechanisms behind premature fruiting in *A. villosum*, and address the agricultural challenges caused by prolonged growth cycles.

## 1. Introduction

*Amomum villosum* Lour. is a perennial herb in the family Zingiberaceae and is widely recognized for its mature fruit, ‘ructus Amomi’, which serves as both a food item and a medicine in traditional Chinese pharmacology. Designated as one of the ‘Four Great Southern Medicines’, it exhibits therapeutic effects including ‘removal of dampness’ to regulate stomach activity, warming of the Middle Jiao to arrest diarrhea, and stabilization of fetal conditions [1]. Under current cultivation practices, this crop relies on small-scale, decentralized farming by individual growers. However, its lengthy growth cycle—approximately 3–4 years from seed germination to first fruiting—results in high initial investment costs and a prolonged revenue cycle, which pose significant challenges to the industry’s sustainable development.

Our research team conducted field surveys and practical agricultural observations and identified a distinct premature fruiting phenomenon in *A. villosum* in which certain *A. villosum* plants began flowering and fruiting within their first year of planting. These plants subsequently entered an initial production phase, entering the reproductive growth stage in the second year, but production failed to reach the full production stage in the following year and achieved high fruit yields by the third year. Compared with typical *A. villosum* varieties, premature fruiting *A. villosum* plants showed early fruiting, early yield production, and early profitability, offering substantial advantages and enhanced potential for agricultural production and a promising opportunity to address the delayed economic returns commonly experienced during the cultivation of this crop. However, breeding research into *A. villosum* faces two significant challenges. First, the absence of screening criteria for premature fruiting traits based on phenotypic/genotypic correlation analysis results in inefficient selection of superior individual plants. Second, premature fruiting lines have heterogeneous genetic backgrounds, and there is currently no comprehensive breeding system for the large-scale production of plants with premature fruiting traits.

According to Zimmerman [2], the growth and reproductive stages of seedlings are divided into the childhood stage (nutritive growth), the transition stage (phase transition from nutritive to reproductive growth), and the adult stage (reproductive growth). The concept of ‘childhood’ also exists for woody plants, spanning the period from seed sowing and sprouting to a plant’s ability to differentiate flower buds, i.e., the process of floral transition. There are three hypotheses regarding this process: 1. ‘Florigen/antiflorigen concept’: Chailakhyan [3] postulated a leaf-derived, phloem-mobile ‘florigen’ that activates floral genes, yet the substance remains unidentified. 2. ‘Nutrient diversion hypothesis’: Sachs & Hackett [4] proposed that flowering is regulated by source/sink relationships. Transition requires the shoot apical meristem to be a strong sink adequately supplied with resources, particularly sucrose, which is considered a prerequisite for flowering. 3. ‘Multifactorial control hypothesis’: this was proposed by Bernier et al. [5,6] and states that the transition to flower formation is determined by a combination of nutrients and various types of hormones and metabolites in the plant, as well as by external photoperiod cues, and so on.

Breeding for early maturity and premature fruiting traits in crops is currently a key subject of study in plant production. To date, there have been no studies on premature fruiting in *A. villosum*; the regulatory mechanisms involving endogenous hormone balance, photoperiodic response, flower formation regulatory pathways, and epigenetic modifications have been more frequently reported for woody cash crops (e.g., *Juglans regia*, *Castanea mollissima*), fruit trees (e.g., *Citrus reticulata*, *Malus pumila*), and the model plant *Arabidopsis thaliana*, which provide a valuable theoretical basis for the study of premature fruiting [7,8,9,10,11]. There is an urgent need to analyze the molecular basis of premature fruiting traits in *A. villosum* and to develop an efficient screening system to accelerate the genetic improvement of this species.

Based on the above background, the primary impetus for investigating the premature fruiting phenomenon in *A. villosum* lies in its significant industrial value and the pressing scientific questions it raises. On the one hand, market demand for *A. villosum* continues to rise, while the impacts of global climate change on crop production are becoming increasingly severe [12]. On the other hand, such an investigation can solve the problem of farmers having no income in the early stages. A comprehensive understanding of the mechanisms governing its growth, development, and yield is essential to ensure the sustainability of fruit supply. However, the current understanding of this phenomenon remains at the observational level, with its underlying biological mechanisms yet to be elucidated and efficient screening methods still lacking. We examined precocious *A. villosum* and typical *A. villosum* plants, screened for the important signaling pathways related to premature fruiting using transcriptome sequencing (RNA-seq) technology, and analyzed the molecular mechanism governing the regulation of premature fruiting, in order to provide a basis for the molecular-assisted breeding of premature fruiting germplasm in *A. villosum*. The ultimate objective is to develop molecular marker-based screening techniques for precocious individuals through in-depth studies of key genes, followed by the breeding and promotion of precocious varieties to support the sustainable development of the *A. villosum* industry.

## 2. Materials and Methods

### 2.1. Plant Material

Seedlings of the variety ‘Yunsha No.8’ were selected from a single source, cultivated at the same time and under the same growth conditions, and transplanted to a breeding base in Mengla County in the Dai Autonomous Prefecture of Xishuangbanna, Yunnan Province, China (21°29′ N, 101°55′ E). Following transplanting, we conducted continuous observation of these plants. Precocious plants initiated reproductive development in the second year and reached full yield capacity by the third year. In contrast, standard genotypes remained strictly vegetative during the first two years and produced their first fruits in the third year. The operational criterion for classification was the presence or absence of flowering and fruit set within the first 2 years after transplanting. Premature fruiting plants (Precocious) and typical plants (CK) were randomly selected for analysis, each with three biological replicates, termed Precocious-1, Precocious-2, and Precocious-3, and CK-1, CK-2, and CK-3. The samples were immediately placed into liquid nitrogen and quickly stored in an ultra-low temperature refrigerator at −80 °C to ensure that the biological properties and related metabolic activities of the samples remained intact to provide a reliable basis for subsequent detection and analysis.

### 2.2. RNA Extraction, Library Construction, and Sequencing

The RNA of the *A. villosum* samples was extracted using an RNAprep Pure Polysaccharide and Polyphenol Plant Total RNA Extraction Kit (Tiangen Biotech Co., Ltd., Beijing, China) and the RNA concentration and absorbance value (A) ratios (A260/A280) at 260 and 280 nm were detected and recorded using a NanoDrop™ OneUV-Vis spectrophotometer (Thermo Fisher Scientific, Madison, WI, USA). The A260/A280 ratios in the test results are all within the range of 1.8 to 2.0. After passing concentration and integrity tests, the cDNA library was submitted to the Beijing Genomics Institution Co., Ltd. (Beijing, China). A cDNA library was constructed and filtered using SOAPnuke (v. 1.4.0), before de novo assembly using Trinity (v. 2.0.6) to obtain clean reads. Tgicl (v. 2.1) was used to cluster and remove redundant transcripts to obtain the Unigenes, and the quality of the assembled transcripts was assessed using the single-copy direct homology database, BUSCO (https://busco.ezlab.org/, accessed on 17 October 2024).

### 2.3. Gene Expression Analysis

The clean reads were compared with genomic sequences using Bowtie (v. 2.4.5), and the expression levels of the genes and transcripts were calculated using RSEM (v. 1.2.8). The differentially expressed genes (DEGs) between the samples were analyzed using the DESeq2 algorithm with *Padj* < 0.05 and |log2FC| ≥ 1. The screened DEGs were enriched and analyzed using the Gene Ontology (GO) and Kyoto Encyclopedia of Genes and Genomes (KEGG) databases, where terms with an adjusted *p*-value < 0.05 were considered statistically significant.

### 2.4. Real-Time Fluorescence Quantitative Polymerase Chain Reaction (RT-qPCR) Analysis of DEGs

The significant DEGs were selected from the RT-qPCR sequencing results. We prioritized the validation of DEGs by exhibiting significantly larger fold changes, as identified in the RNA-seq results. Specific primers were designed using Primer Premier (v. 6.0) and synthesized by the Beijing Liuhe Huada Gene Technology Co. (Beijing, China). The primer sequences are shown in Table 1. Amplification was performed using TB Green Premix Ex Taq Ⅱ (TaKaRa, Kusatsu, Shiga, Japan) and the CFX96 Fluorescence Quantitative PCR Instrument (Bio-Rad), using a two-step method with the following amplification conditions (reference manual): pre-denaturation at 95 °C for 30 s, 95 °C for 5 s, and 60 °C for 30 s. Forty cycles were performed, with three technical replicates per sample. Actin was used as the internal reference gene, and the relative expression was calculated using the 2^−ΔΔCt^ method.

## 3. Results

### 3.1. Analysis of RNA-Seq Libraries

A total of 29.0 Gb of data were sequenced using the DNBSEQ^TM^ platform. After assembly and redundancy removal, 115,965 unigenes were obtained, with total length, average length, N50, and GC contents of 158,687,045 bp, 1368 bp, 2272 bp, and 43.26%, respectively (Table 2). The BUSCO assessment results are presented in Supplementary Figure S1.

### 3.2. Differential Expression Analysis of Transcripts

*Padj* and log2FC were used to screen for DEGs, with screening conditions of *Padj* < 0.05 and |log2FC| ≥ 1. The analysis of the DEGs showed a total of 1545 DEGs, including 559 upregulated DEGs and 986 downregulated DEGs.

### 3.3. GO Functional Annotation and Enrichment Analysis of DEGs

**Figure 1:**
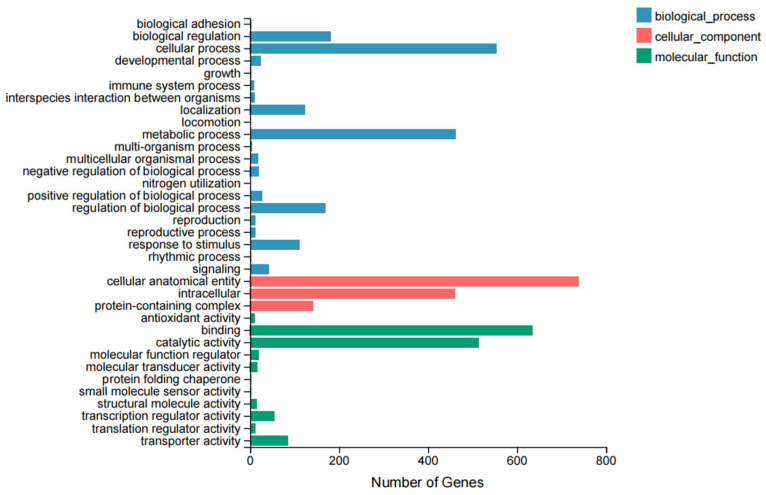
GO functional annotation classification of DEGs.

The GO database is categorized into three functional classes: molecular function, cellular component, and biological process. Within each major category, there are a total of 35 subcategories at various levels. Figure 1 shows the GO annotation classification results of the DEGs identified in this study. The specific information is presented in Supplementary Table S1. In the biological process category, the DEGs were mainly concentrated in cellular process, metabolic process, and biological regulation; in the cellular component category, DEGs were distributed between cellular anatomical entity, and intracellular and antioxidant activity; and in the molecular function category, the DEGs were mainly concentrated in binding and catalytic activity.

**Figure 2:**
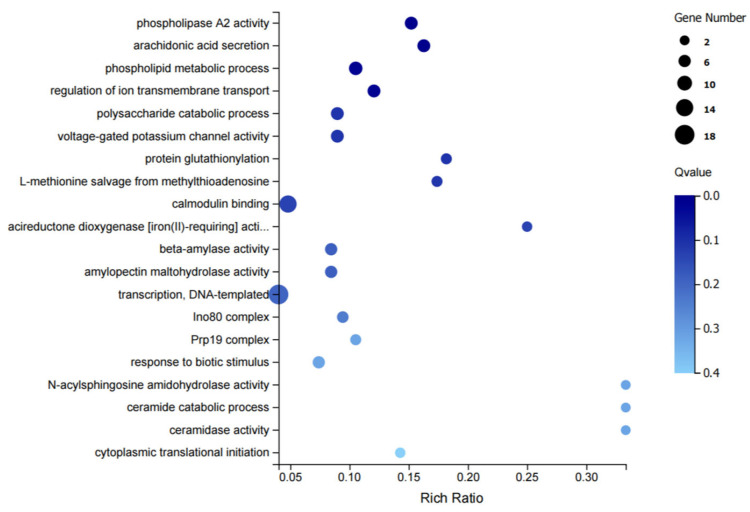
GO enrichment analysis of DEGs.

GO enrichment analysis of these DEGs resulted in a total of 1136 pathways. Figure 2 shows the top 20 pathways, which mainly comprise phospholipase A2 activity (GO: 0004623), arachidonic acid secretion (GO: 0050482), phospholipid metabolic process (GO: 0006644), regulation of ion transmembrane transport (GO: 0034765), polysaccharide catabolic process (GO: 0000272), and other pathways.

### 3.4. KEGG Functional Annotation and Enrichment Analysis of DEGs

**Figure 3:**
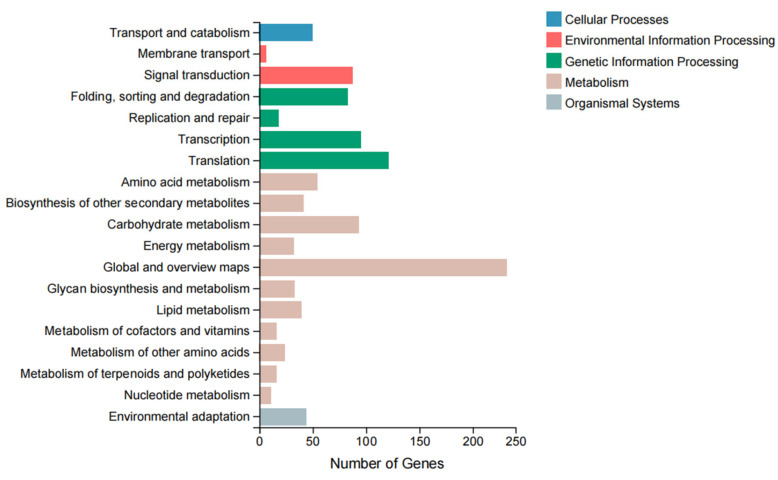
KEGG signaling pathway classification of DEGs.

The KEGG metabolic gene pathways are divided into five categories: Cellular Processes, Environmental Information Processing, Genetic Information Processing, Metabolism, and Organismal Systems. Each category is further subdivided, and the DEGs identified were annotated into 19 of these pathways. Figure 3 shows the classifications of the KEGG pathway annotations for the discovered DEGs. Among them, the most annotated secondary pathways were Global and Overview Maps, Translation, Transcription, and Carbohydrate Metabolism.

**Figure 4:**
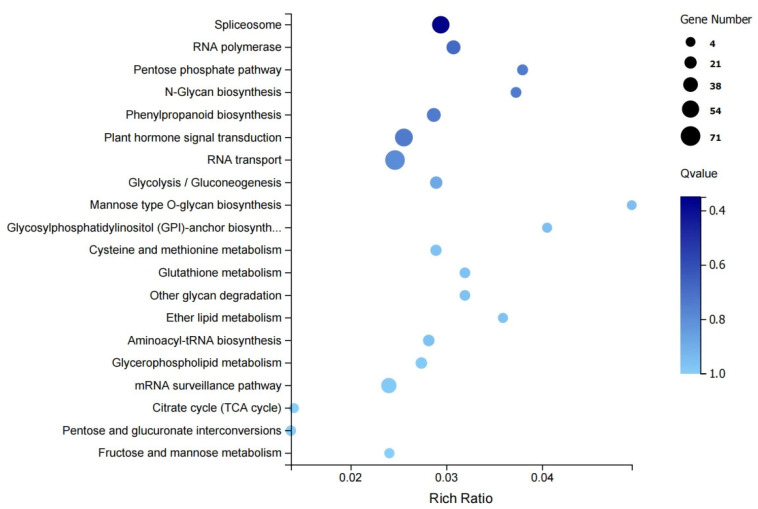
KEGG enrichment analysis of DEGs.

The KEGG enrichment analysis of the DEGs revealed a total of 114 metabolic pathways, of which the 20 major pathways are shown in Figure 4, including the spliceosome (ko03040), the RNA polymerase pathway (ko03020), the pentose phosphate pathway (ko00030), plant hormone signal transduction (ko04075), and mannose type O-glycan biosynthesis (ko00515).

### 3.5. Validation of DEGs Using RT-qPCR

**Figure 5:**
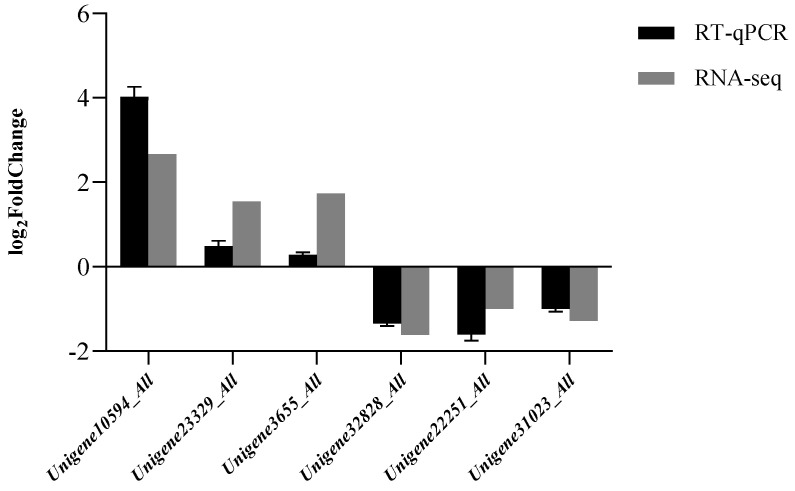
RT-qPCR verification of RNA-seq results.

Six DEGs were randomly selected for RT-qPCR analysis, comprising three upregulated genes (*Unigene10594_All*, *Unigene23329_All*, and *Unigene3655_All*) and three downregulated genes (*Unigene32828_All*, *Unigene22251_All*, and *Unigene31023_All*). The relative expression of the six DEGs and the transcriptome sequencing results are shown in Figure 5. The trend in the expression of these six genes was consistent with the trend in the transcriptome sequencing results, and there was a strong correlation between RT-qPCR and RNA-seq (correlation coefficient *R* = 0.86943), indicating the reliability of the transcriptome sequencing results.

## 4. Discussion

In this study, the KEGG enrichment analysis showed that 59 DEGs were enriched in the plant hormone signal transduction (ko04075) pathway (Figure 4). These DEGs are listed in the Supplementary Materials (Table S2). Plant hormones play important roles in various biological processes, including plant growth, development, flowering, and fruiting. Tong et al. [13] used gas–liquid chromatography–electron capture and hydrogen flame ionization techniques to detect the levels of endogenous cytokinins, gibberellins, growth hormones, and abscisic acid in the female flower buds of three-year-old early- and late-fruiting *Juglans regia* tree types, and the results show that changes in endogenous hormones have an important impact on flower bud differentiation.

Further analysis revealed that the DEGs in this pathway are associated with genes encoding key components of the auxin (IAA) signaling pathway, including *Auxin 1* (*AUX1*), *Transport Inhibitor Response 1* (*TIR1*), AUX/IAA, *Auxin Response Factor* (*ARF*), *Gretchen Hagen 3* (*GH3*), and *Small Auxin Up RNA* (*SAUR*). These genes play crucial roles in auxin recognition, signaling, and responses. For instance, *TIR1* mediates the degradation of AUX/IAA proteins by transmitting early auxin signals, while *ARF* regulates downstream target gene expression to influence organ development and the flowering process, thereby affecting overall plant growth and development. IAA has been shown to inhibit flowering in *Citrus* [14] and *Pharbitis nil* [15]. The *Cytokinin Response 1* (*CRE1*) and *Type-B Arabidopsis Response Regulator* (*B-ARR*) genes are central to the cytokinin (CTK) signaling pathway. *CRE1*, a cytokinin receptor, perceives cytokinin signals and initiates downstream signaling cascades. CTK is involved in cellular proliferation and differentiation and delays senescence in flowering plants. Sapkota et al. [16] reported that the CTK content of apple is significantly higher at the budding stage than during the bud dormancy and early eco-dormancy stages. Early-flowering apple varieties exhibit increased CTK content up to 12 days earlier than late-flowering varieties, suggesting that CTK promotes budding and flowering. In contrast, lower CTK levels are required for bud differentiation in *Paphiopedilum hirsutissimum* [17]. DELLA and transcription factor (TF) genes are key components of the gibberellin (GA) signaling pathway. GAs promote the ubiquitination and degradation of DELLA proteins, releasing their inhibitory effect on TFs, which then activate flowering-related genes. The GA pathway is a critical regulator of plant growth and development and plays a significant role in the regulation of flowering. Notably, low GA concentrations promote flowering, while high concentrations inhibit it [18]. The *Protein Phosphatase 2C* (*PP2C*), *Snf1-Related Protein Kinase 2* (*SnRK2*), and *Aba-Responsive Element Binding Factor* (*ABF*) genes are integral to abscisic acid (ABA) signaling. *PP2C* acts as a negative regulator in ABA signaling, *SnRK2* acts as a positive regulator, and *ABF* is a TF. ABA levels rise under adverse conditions such as drought and high temperatures and interact with *PP2C*, *SnRK2*, and *ABF* to influence flowering. In *Pyrus pyrifolia* [19], ABA dynamics are synchronized with the flowering process. Wen et al. [20] observed higher ABA levels in late-flowering *Prunus mume* varieties compared with early-flowering ones. Similarly, Sapkota et al. [16] found that high ABA levels during dormancy correlate with later flowering in apples. The *Sativa Stress-Induced Mitogen-Activated Protein Kinase Kinase* (*SIMKK*), *MAP Kinase 6* (*MPK6*), and *Ethylene Insensitive 3* (*EIN3*) genes are part of the ethylene (ETH) signaling pathway. *SIMKK* and *MPK6* participate in the MAP kinase cascade, while *EIN3* is a key TF. Ethylene inhibits vegetative growth by increasing IAA oxidase activity and reducing IAA levels, decreases GA3 and CTK activities, and induces flower bud differentiation [21,22]. In *Mangifera indica*, ethylene glycol treatment increases the zeatin riboside (ZR) and ABA contents, increases the ABA/GA3 and ZR/GA3 ratios, and promotes flower bud differentiation [23]. The *Brassinosteroid Insensitive 1* (*BRI1*) and *Brassinosteroid Insensitive 2* (*BIN2*) genes are central to the brassinosteroid (BR) signaling pathway. *BRI1*, a receptor kinase, perceives BR signals and initiates downstream signaling, while *BIN2* is a protein kinase that negatively regulates BR signaling. BR is directly involved in photomorphogenesis and interacts with the photoperiodic pathway, another key pathway influencing flowering. As the retarded growth in BR-insensitive and BR-deficient mutants causes a strong delay in days to flowering, BR signaling has been thought to promote the floral transition in *Arabidopsis* [24]. BRs regulate tomato early flowering through the interaction between *SlFRIGIDA-LIKE (SlFRLs)* and *SlBIN2* [25]. The *Jasmonate Resistant 1* (*JAR1*), *Jasmonate Zim-Domain Proteins* (*JAZ*), and *MYC2* genes are key components of the jasmonic acid (JA) signaling pathway. *JAR1* is involved in JA synthesis and metabolism, *JAZ proteins* are negative regulators, and *MYC2* is a downstream TF. JA plays a crucial role in the physiological and morphological differentiation of flowering. In a study comparing two apple varieties with different flowering times, Jasmonoyl-Isoleucine (JA-Ile) was significantly upregulated in late-flowering varieties during the bud stage, suggesting a link between higher JA levels and late flowering [16]. The key flowering gene *CONSTANS* (*CO*) represses JA expression, promoting flower senescence [26]. The NPR1 regulatory protein (NPR1) and *TGACG Motif-Binding Factor* (*TGA)* genes are central to the salicylic acid (SA) signaling pathway. NPR1 is a key regulatory protein, and *TGA* is a TF.

**Figure 6:**
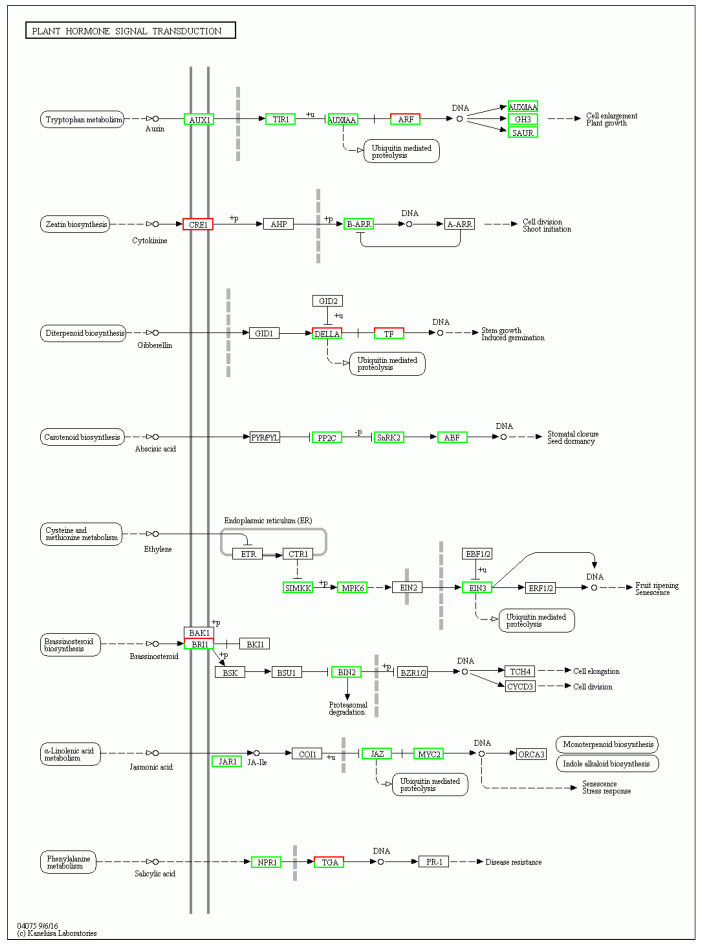
Plant hormone signal transduction pathway. The genes marked with green/red boxes are the differentially expressed genes identified in this study and are also the preliminary candidate genes.

The phytohormone signaling pathway plays a complex and delicate role in the regulation of flowering, and different hormones interact and regulate each other, forming a subtle regulatory network. The above results suggest that premature fruiting in *A. villosum* is jointly regulated by multiple hormones (Figure 6).

The process of plant flowering and fruiting is extremely energy-consuming. Only when nutrient accumulation reaches a certain level can a plant shift from non-reproductive growth to reproductive growth. The phospholipid metabolism (GO: 0006644) (Table S3) suggested by the GO enrichment analysis, the carbohydrate metabolism pathway suggested by the KEGG functional annotations, and the pentose phosphate pathway (ko00030) (Table S4, Figure S2) suggested by the KEGG enrichment analysis all play important roles in supplying a plant with energy. In addition to being related to phytohormone regulation, premature fruiting in plants is a complex process that may be caused by the synergistic regulation of multiple pathways. In addition, the GO enrichment analysis results suggested that the arachidonic acid secretion pathway may also be involved. Arachidonic acid (GO: 0050482) is a mediator of phytohormone signaling, plays an important role in plant stress tolerance, and also interacts with hormones such as JA and SA to regulate plant growth and development.

However, research on this phenomenon is still incomplete. Future studies should build upon these findings to conduct further analysis and screening, identifying key genes involved in premature fruiting. Experimental validation—including gene cloning, functional characterization, expression pattern analysis, and gene network construction—is essential to confirm these key genes’ roles. This will provide a comprehensive explanation of the premature fruiting mechanism. Successful elucidation of the underlying molecular mechanisms and their application in production could significantly address current challenges in the promotion of *A. villosum*. On this basis, molecular markers can also be developed for screening and identifying the premature fruiting single plant.

## 5. Conclusions

A distinct premature fruiting phenomenon was identified in *A. villosum,* in which certain *A. villosum* plants began flowering and fruiting within their first year of planting. In this study, we used RNA-seq and bioinformatic analyses to explore the regulatory mechanisms underlying premature fruiting in *A. villosum* by identifying the key DEGs and metabolic pathways. The sequencing generated 29.0 Gb of clean data, and 115,965 unigenes were identified, with an average length of 1368 bp. Based on the sequencing results, 1545 DEGs were identified, including 559 upregulated and 986 downregulated genes. GO analysis of the DEGs revealed their involvement in cellular processes, cellular anatomical entities, and binding. GO enrichment highlighted 1136 pathways associated with phospholipase A2 activity, arachidonic acid secretion, the phospholipid metabolic process, the regulation of ion transmembrane transport, and the polysaccharide catabolic process. KEGG pathway analysis allocated the DEGs to 19 metabolic pathways, including global overview maps, translation, transcription, and carbohydrate metabolism. KEGG enrichment identified a further 114 pathways, predominantly spliceosome, RNA polymerase, pentose phosphate, plant hormone signal transduction, and mannose type O-glycan biosynthesis. Our findings demonstrate that phytohormone signaling, carbohydrate and lipid metabolism, and polysaccharide degradation play pivotal roles in the control of premature fruiting in *A. villosum*. Six randomly selected DEGs were validated using RT-qPCR, and the results corroborated the transcriptome data, confirming their reliability. Future research directions will focus on three primary objectives:

1. Identification and functional characterization of key genes: Further investigation will target the discovery of critical genetic determinants and the elucidation of their mechanistic roles in regulating premature fruiting.
2. Alternative splicing events need to be included in future experimental research, which is also one of the foundations for developing molecular markers.
3. Development of molecular markers for precision breeding: The establishment of molecular marker-assisted selection protocols will enable the efficient screening of premature fruiting individuals, thus accelerating the breeding and dissemination of elite premature fruiting germplasm lines.

In conclusion, this study enriches the understanding of the basic biology of this species; promotes research into comparative genomics, genetic mapping, and gene function; lays a solid foundation for the study of the molecular mechanisms behind premature fruiting in *A. villosum*; and addresses the agricultural challenges caused by prolonged growth cycles.

## Figures and Tables

**Table 1:** Primers used for RT-qPCR validation.

Gene Name	Forward Primer Sequences (5′-3′)	Reverse Primer Sequences (5′-3′)
*Unigene10594_All*	ACTCATCGTGCCAGTGCTCCA	GCGTTGGTCTGCTCCATCTTCC
*Unigene23329_All*	ACGGACCCTTCCTCAGGCAAT	TGGCAGCAGGAGCAGTAGCA
*Unigene3655_All*	GGAGAACGCATTGGCAGTGTCA	TGGGTGGTTCAGAGGCAACAGA
*Unigene32828_All*	GTCTCTGTACCGTGCCGTCATC	GCTGTCCGACTGGTGCTTGTAG
*Unigene22251_All*	CACGCCACCAGGAAGTTGTCAG	TTCCCTCGTCCCTCTTCTTGCC
*Unigene31023_All*	CCGCCGTTGATAGAGGACTTCC	GTCTTCGTGGACACTCTGCCTT
*Actin*	CGCATTGACGACCTCCAGTG	TCTTCACCGCATGTGACAATCC

**Table 2:** Statistical results of RNA-seq data analysis.

Material	Total Raw Read (M)	Total Clean Read (M)	Clean Read Q30 (%)	Clean Read Ratio (%)	Total Mapping Gene Ratio (%)	Uniquely Mapping Gene Ratio (%)	N50 (bp)
CK-1	43.82	42.29	90.84	96.51	83.35	22.41	1938
CK-2	45.57	43.41	91.20	95.26	84.31	23.74	1719
CK-3	43.82	42.35	90.99	96.64	85.43	25.38	1681
Precocious-1	40.03	38.24	91.01	95.55	81.48	21.91	1913
Precocious-2	43.82	42.05	90.82	95.95	82.35	23.89	1743
Precocious-3	45.57	43.41	91.19	95.24	83.69	22.11	1931

## Data Availability

Data are contained within this article.

## Supplementary Materials

The following supporting information can be downloaded at: https://www.mdpi.com/article/10.3390/biology14070883/s1. Figure S1: BUSCO assessment results of the *Amomum villosum* genome assembly. Figure S2: Pentose phosphate pathway (ko00030). The genes marked with green/red boxes are the differential genes identified in this study and are also the preliminary candidate genes. Table S1: Specific results of the GO functional annotation classification of DEGs. Table S2: Annotation of DEGs in the plant hormone signal transduction (ko04075). Table S3: Annotation of DEGs of phospholipid metabolism (GO: 0006644). Table S4: Annotation of DEGs in the pentose phosphate pathway (ko00030).

## Abbreviations

The following abbreviations are used in this manuscript:

*A. villosum.*	*Amomum villosum* Lour.
RNA-seq	Transcriptomic Sequencing
DEGs	Differentially Expressed Genes
GB	Gigabases
GO	Gene Ontology
KEGG	Kyoto Encyclopedia of Genes and Genomes
RT-qPCR	Real-Time Fluorescence Quantitative Polymerase Chain Reaction
IAA	Auxin
*AUX1*	*Auxin 1*
*TIR1*	*Transport Inhibitor Response 1*
*ARF*	*Auxin Response Factor*
*GH3*	*Gretchen Hagen 3*
*SAUR*	*Small Auxin Up R**NA*
CTK	Cytokinin
*CRE1*	*Cytokinin Response 1*
*B-ARR*	*Type-B Arabidopsis Response Regulator*
TF	Transcription Factor
GA	Gibberellin
*PP2C*	*Protein Phosphatase 2C*
*SnRK2*	*Snf1-Related Protein Kinase 2*
*ABF*	*Aba-Responsive Element Binding Factor*
ABA	Abscisic Acid
*SIMKK*	*Sativa Stress-Induced Mitogen-Activated Protein Kinase Kinase*
*MPK6*	*MAP Kinase 6*
*EIN3*	*Ethylene Insensitive 3*
ETH	Ethylene
ZR	Zeatin Riboside
*BRI1*	*Brassinosteroid Insensitive 1*
*BIN2*	*Brassinosteroid Insensitive**2*
BR	Brassinosteroid
*SlFRLs*	*SlFRIGIDA-LIKE*
*JAR1*	*Jasmonate Resistant 1*
*JAZ*	*Jasmonate Zim-Domain Proteins*
JA	Jasmonic Acid
JA-Ile	Jasmonoyl-Isoleucine
CO	CONSTANS
NPR1	NPR1 Regulatory Protein
*TGA*	*TGACG Motif-Binding Factor*
SA	Salicylic Acid
