# Supplementary figures and images for: Transcriptome Sequencing-Based Analysis of Premature Fruiting in Amomum villosum Lour
Source: Biology (Basel). 2025 Jul 18;14(7):883. doi: 10.3390/biology14070883 (PMC12293058; doi:10.3390/biology14070883)

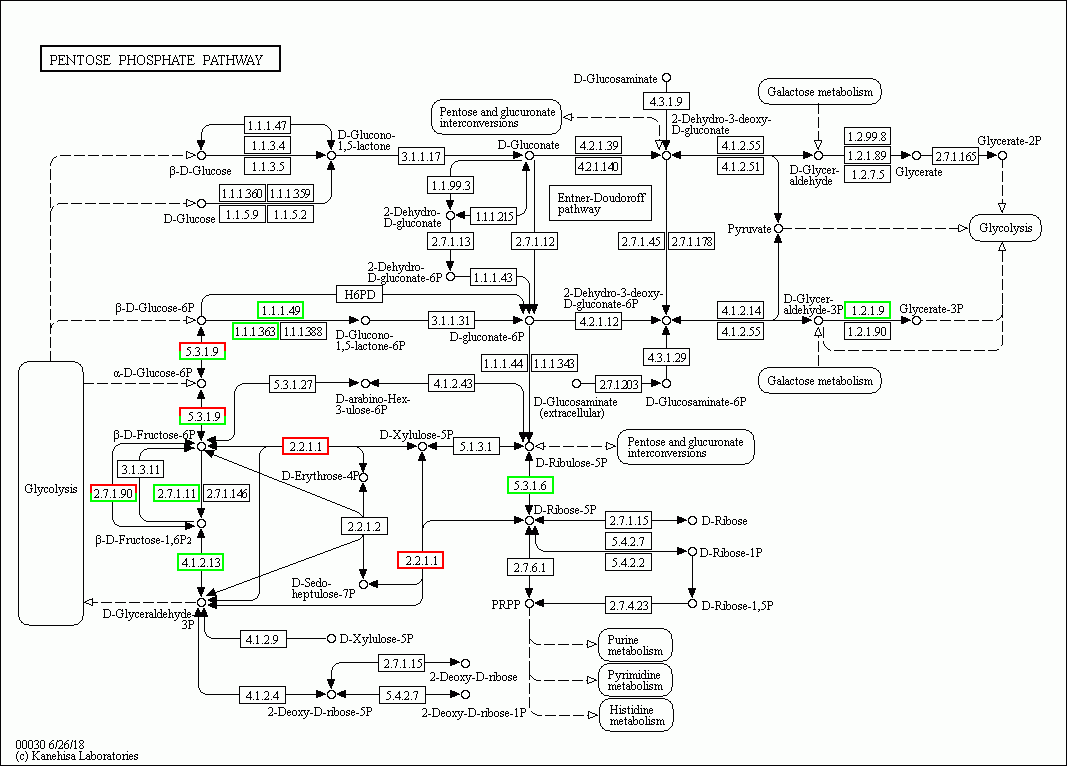

Supplement: Supplementary file 1 [file biology-14-00883-s001.zip › biology-3740820-Figure S2.png]

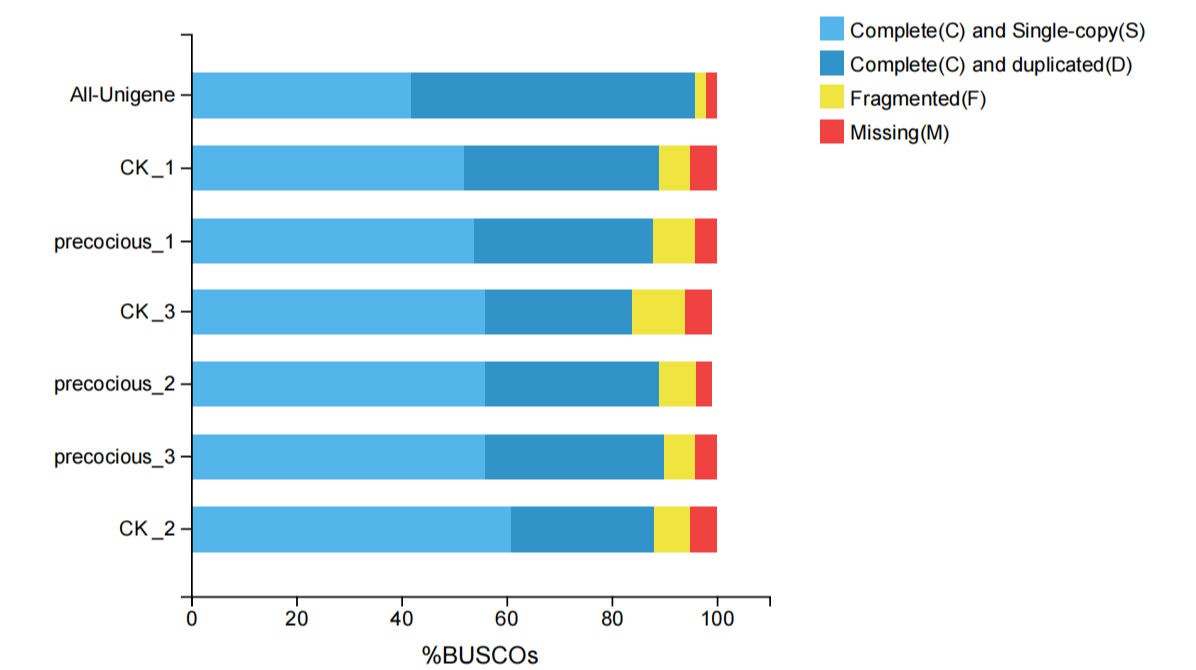

Supplement: Supplementary file 1 [file biology-14-00883-s001.zip › biology-3740820-Figure S1.png]
